# The role of primary inguinal surgical debulking for locally advanced penile cancer followed by reconstruction with myocutaneous flap

**DOI:** 10.1590/S1677-5538.IBJU.2021.0169

**Published:** 2021-06-10

**Authors:** Leandro Koifman, Daniel Hampl, Marcio Ginsberg, Rodrigo Barros de Castro, Nelson Koifman, Paulo Ornellas, Antonio Augusto Ornellas

**Affiliations:** 1 Hospital Municipal Souza Aguiar Serviço de Urologia Rio de JaneiroRJ Brasil Serviço de Urologia, Hospital Municipal Souza Aguiar, Rio de Janeiro, RJ, Brasil;; 2 Instituto Nacional de Câncer Departamento de Urologia Rio de JaneiroRJ Brasil Departamento de Urologia, Instituto Nacional de Câncer - INCA, Rio de Janeiro, RJ, Brasil

**Keywords:** Penile Neoplasms, Reconstructive Surgical Procedures, Myocutaneous Flap

## Abstract

**Purpose:**

To evaluate surgical complications and oncological outcomes of patients submitted to primary radical inguinal surgical debulking (PRISD) and myocutaneous pediculate flap reconstruction (MPFR) for locally advanced penile cancer (PC).

**Materials and Methods:**

Forty-two patients with ulcerated and/or fixed bulky inguinal masses underwent unilateral or bilateral PRISD with MPFR. Tensor fascia lata flap (TFL) was the standard of care for all patients. Additional use of the gracilis flap (GF) was carried out when necessary. Contra-lateral radical inguinal lymphadenectomy (RIL) was conduced when PRISD was performed unilaterally. Surgical complications were analyzed and stratified into minor and major according to the Bevan-Thomas classification. Adjunctive treatments were assessed and oncological outcomes analyzed.

**Results:**

Of the 42 patients evaluated, 10 (23.8%) underwent bilateral PRISD and 32 (76.2%) unilateral PRISD with contra-lateral RIL, totaling 84 lymphadenectomies. A total of 62 MPFRs were performed, 52 with TFL and 10 with GF. A total of 53 complications were identified, 49 related to PRISD with MPFR and 4 to RIL. Adjuvant chemotherapy was carried out in 16 patients. Median follow-up was 10.8 months with a median overall survival (OS) of 14.0 months against 6.0 months (p=0.006) for patients submitted to PRISD with adjuvant chemotherapy in relation to surgery alone.

**Conclusions:**

PRISD alone for advanced loco-regional PC is unlikely to promote long-term survival, although it can lead to temporary local control of the disease. Despite the feasibility of the procedure, it is related to high incidence of complications. Surgical treatment with adjuvant chemotherapy is associated with improved OS.

## INTRODUCTION

Penile cancer (PC) is a rare neoplasm with low incidence in developed countries, in contrast with high incidence in developing countries, clearly indicating the disease's association with local economic conditions (1, 2).

Patients with PC tend to seek medical care belatedly, with about 15-50% of them presenting symptoms for more than one year. This delay is mainly attributed to embarrassment, guilt, fear, ignorance, personal neglect and difficulty of access to the public health system, especially in developing countries (2, 3). The delay in diagnosis and treatment of these patients can drastically reduce survival.

The presence and extent of inguinal metastases are the most important prognostic factors related to survival of patients with squamous cell carcinoma of the penis (1, 4-7).

Approximately 0-14% of patients with PC initially present locally advanced disease, with bulky metastatic lesions in the inguinal lymph nodes. Therapeutic options at this stage of the disease are usually scarce, limited to palliative radiotherapy and chemotherapy. Untreated, these patients have a mortality rate up to 90% in two years (8, 9).

Patients in this clinical stage suffer the progressive course of the disease, often associated with skin necrosis, chronic infection of the tumor site, pain, fetor, sepsis, bleeding related to tumor erosion into vascular structures, and cachexia, leaving the patient bedridden, with low quality of life and miserable demise.

In the past, these cases were considered beyond the possibility of surgical therapy, but in order to reintegrate these patients into society and provide them with a more dignified end of life with their families, cytoreductive surgeries are now often performed with palliative or curative intent. Despite the unclear role of surgery in the management of locally advanced disease, when performed it leads to large wound defects, with invariable necessity of surgical techniques for tissue reconstruction. In this context, there is currently no consensus in the international literature, based on strong available data regarding the best practice for treating locally advanced disease, considering long-term benefits versus complications, especially in the setting of primary surgery.

The aim of this study was to evaluate the complications and oncological outcomes of primary radical inguinal surgical debulking (PRISD) with myocutaneous pediculate flap reconstruction (MPFR) as first-line treatment for local advanced PC.

## MATERIAL AND METHODS

### Patient Characteristics

Between January 2010 and December 2018, 42 patients with stage IV PC were admitted to our facility and prospectively evaluated. Median patient age was 51.5 years (range 23 to 92). Only patients with ulcerated and/or fixed bulky inguinal masses, without previous inguinal node treatment, were included in this study. All patients underwent biopsy of the primary lesion for diagnostic confirmation. Patients were clinically evaluated for inguinal and visceral metastasis based on physical examination of the inguinal region and computerized tomography (CT) of the chest, abdomen and pelvis. Pathological material was reviewed and all tumors were histologically classified based on Broder's system. The presence of extranodal disease extension of the specimens obtained after PRISD was also evaluated. A single pathologist was responsible for reviewing the specimens. The clinical and pathological staging was done according to the TNM classification system 2002. The time elapsed between treatment of the primary lesion and inguinal dissection was evaluated. We also evaluated the operative time required for each procedure and the length of hospital stay.

Patients were categorized pre-operatively according to the Eastern Cooperative Oncology Group (ECOG) Performance Status Classification (Supplementary Material) (10).

All patients underwent unilateral or bilateral PRISD according to inguinal lymph node status. All patients systematically underwent contra-lateral standard radical inguinal lymphadenectomy (RIL) according to the technique described by Ornellas et al. (11), when PRISD was performed unilaterally. None of the patients underwent pelvic lymphadenectomy. All patients underwent MPFR by a plastic surgery team, according to the tissue defect produced by the inguinal surgical debulking. Use of the myocutaneous tensor fascia lata flap (TFL) was the standard of care for all patients. Additional use of the myocutaneous gracilis flap (GF) occurred when necessary for complete coverage of the tissue defect produced by the lymphadenectomy.

After hospital discharge, patients were followed as outpatients monthly for the first three months and then every three months.

All patients provided informed consent and our institutional review board approved the study (IRB 3805). Medical assistants performed data collection during perioperative and outpatient follow-up.

### Surgical Procedure

**Figure 1:**
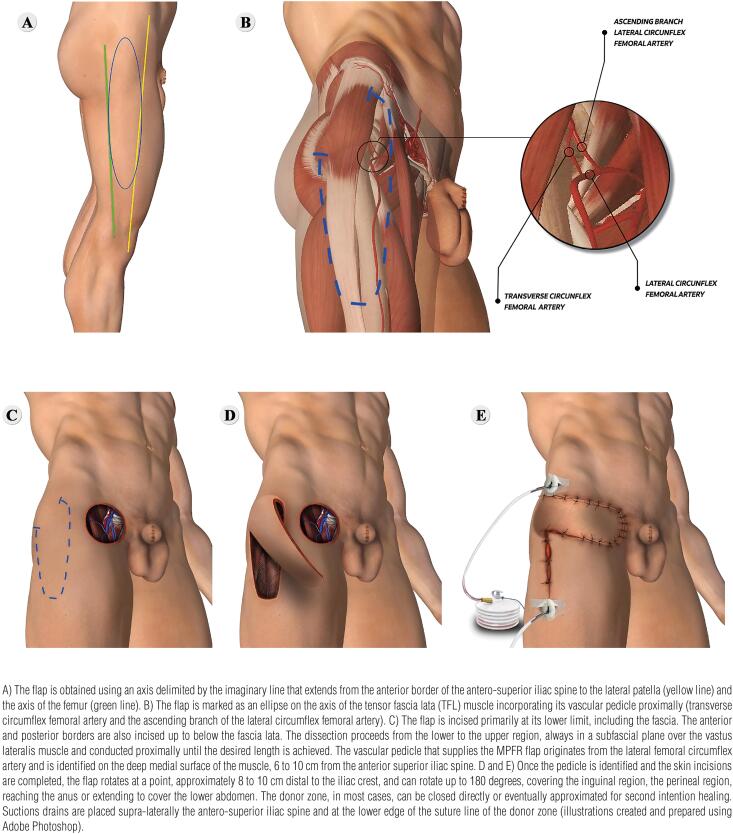
After primary radical inguinal surgical debulking (PRISD) is concluded, the patient is prepared and placed in the supine position for myocutaneous pediculate flap reconstruction (MPFR).

The patient is placed supine with legs fixed in moderate external rotation with operatory field prepared with 2% chlorhexidine in 70% isopropyl alcohol. A circular incision is made, with a 2cm safety margin, encompassing the metastatic inguinal mass composed of adhered or ulcerated skin, subcutaneous cell tissue and lymph nodes infiltrated by the tumor. Any other structure of the perineum, abdomen or inguinal region that is infiltrated by the tumor must be resected en bloc with surgical specimen. The fascia covering the sartorius and long adductor muscles is incised and removed together with the tumor. The saphenous vein crossing and its tributary branches are identified and sectioned. The femoral vessels are left clean until the apex of the femoral triangle. Figure-1 illustrates MPFR using TFL for the wound defect coverage.

### Postoperative Care

All patients received prophylactic antibiotic therapy, which was initiated at anesthetic induction and maintained for 24 hours. We used first-generation cephalosporins in most cases, according to the guidelines of our committee to control hospital infections. In specific cases, cultures were performed of the ulcerated inguinal metastasis tissue and patients were treated in accordance with the result for 14 days. All patients were restricted to bed rest for three days with ambulation starting on postoperative day 4, when possible. Low molecular weight heparin was prescribed for all patients and discontinued after the onset of ambulation. Suction drains were removed after the output was less than 50mL in 24 hours.

### Complications

Complications related to PRISD with MPFR and RIL were analyzed and stratified as minor or major based on the definitions of Bevan-Thomas et al. (12).

### Oncological Outcome Analysis

After hospital discharge, all patients were referred to the clinical oncology department for adjuvant treatment evaluation. Patients were analyzed for social and family reintegration, type of adjuvant oncological treatment, tumor recurrence, disease progression, resection margin status, disease-specific mortality (DSM) and local oncological control. Overall survival (OS) and disease free survival (DFS) analysis was performed according to the Kaplan-Meyer and log-rank tests, using the software IBM SPSS® Statistics version 20.

Surgical satisfaction was measured on a five-point scale, ranging from very unsatisfied to very satisfied, via a questionnaire completed separately by the patient and a family member during the first follow-up appointment.

## RESULTS

### Patients

All patients had squamous cell carcinoma of the penis and extranodal metastatic disease extension in the specimens obtained after PRISD procedure. Table-1 lists primary tumor pathological characteristics with clinical and pathological lymph node status. Of the 42 patients evaluated, 10 (23.8%) underwent bilateral PRISD and 32 (76.2%) unilateral PRISD with contra-lateral RIL, totaling 84 lymphadenectomies, 52 (61.9%) PRISDs and 32 (38.1%) RILs. All PRISD procedures produced large wound defects, requiring reconstructive plastic surgery. A total of 62 MPFRs were performed, 52 involving TFL and 10 GF. Average hospital stay was 15.8 days (range 10 to 58). Average operative time for bilateral PRISD with bilateral MPFR was 337 minutes. Average time for unilateral PRISD with MPFR and contra-lateral RIL was 254 minutes. Time from primary tumor treatment to inguinal dissection was 2 to 4 weeks in 9 patients (21.4%) and 24 to 52 weeks in 5 patients (11.9%), whose follow-up was missed after primary tumor treatment, while in 28 (66.7%) patients, the two procedures were performed simultaneously. All patients were staged through CT of the chest, abdomen and pelvis: 1 patient (2.4%) suffered from pulmonary metastasis and enlarged pelvic lymph nodes, 1 patient (2.4%) presented enlarged pelvic and retroperitoneal lymph nodes besides bulky cervical tumor, and 4 patients (9.5%) suffered from slightly enlarged pelvic lymph nodes (ranging from 1 to 2cm). In the remaining 36 patients (85.7%), CT revealed no visceral metastasis or pelvic lymphadenopathy.

**Table 1:** Clinical and pathological characteristics of primary penile tumor and lymph node status in 42 patients undergoing bilateral PRISD and unilateral PRISD with contralateral RIL.

		No. pT2 (%)	No. pT3 (%)	No. pt4 (%)
**Grade**		26 (61.9)	10 (23.8)	6 (14.3)
	0	5 (11.9)	1 (2.4)	1 (2.4)
	2	12 (28.6)	7 (16.6)	3 (7.1)
	3	9 (21.4)	2 (4.8)	2 (4.8)
**Lymphovascular Invasion**		26 (61.9)	10 (23.8)	6 (14.3)
	Present	17 (40.5)	6 (14.3)	5 (11.9)
	Absent	9 (21.4)	4 (9.5)	1 (2.4)
**Clinical lymph node status**		26 (61.9)	10 (23.8)	6 (14.3)
	cN3	26 (61.9)	10 (23.8)	6 (14.3)
**Pathological lymph node status**		26 (61.9)	10 (23.8)	6 (14.3)
	pN3 (Extra nodal disease extension)	26 (61.9)	10 (23.8)	6 (14.3)

PRISD = primary radical inguinal surgical debulking; RIL = radical inguinal lymphadenectomy

### Complications

A total of 53 complications were identified in this study, 49 related to 52 PRISD with 62 MPFR, of which 40 (81.6%) were minor and 9 (18.4%) major. Only 4 (12.5%) minor complications were associated with 32 RILs. All minor complications resulting from PRISD with MPFR and RIL were treated conservatively. Of the 9 major complications, 2 wound infections were treated with specific intravenous antibiotics and 1 abscess was treated through drainage and intravenous antibiotics. Both patients presented complete resolution of the infectious condition. Two lymphoceles were treated with ultrasound-guided puncture and 2 cases of flap necrosis were treated with surgical debridement with maintenance of flap viability. Two patients developed sepsis in the immediate postoperative period, with evolution to death. Table-2 lists the complication rates and types in patients submitted to PRISD with MPFR and RIL.

**Table 2:** Complications related to 52 PRISD with 62 MPFR (52 TFL and 10 GF) and 32 RIL.

		Complications (%)		
Complication		PRISD + MPFR		RIL
		TFL	GF	
**Minor**		**37 (71.1)**	**3 (30)**	**4 (12.5)**
	Wound infection	1 (2.1)	0 (0)	0 (0)
	Seroma	3 (5.7)	0 (0)	1 (3.1)
	Leg edema trace	0 (0)	N/A	2 (6.25)
	Leg edema +1	7 (13.4)	N/A	0 (0)
	Leg edema +2	5 (9.6)	N/A	0(0)
	Wound dehiscence	11 (21.1)	3 (30)	N/A
	Skin edge necrosis	6 (11.5)	0 (0)	1 (3.1)
	Scrotal edema	4 (7.7)	N/A	N/A
**Major**		**9 (17.3)**	**0 (0)**	**0 (0)**
	Wound infection + intavenous antibiotics	2 (3.8)	0 (0)	0 (0)
	Lymphocele + intervention	2 (3.8)	0 (0)	0 (0)
	Flap necrosis/treatment	2 (3.8)	0 (0)	0 (0)
	Wound abscess/cellulitis	1 (2.1)	0 (0)	0 (0)
	Sepsis/death	2 (3.8)	N/A	N/A
**Total No. (%)**		**46 (88.4)**	**3 (30)**	**4 (12.5)**

PRISD = primary radical inguinal surgical debulking; MPFR = myocutaneous pediculate flap reconstruction; TFL = tensor fascia lata flap; GF = gracilis flap; N/A = not applicable.

### Oncological Outcomes

**Figure 2:**
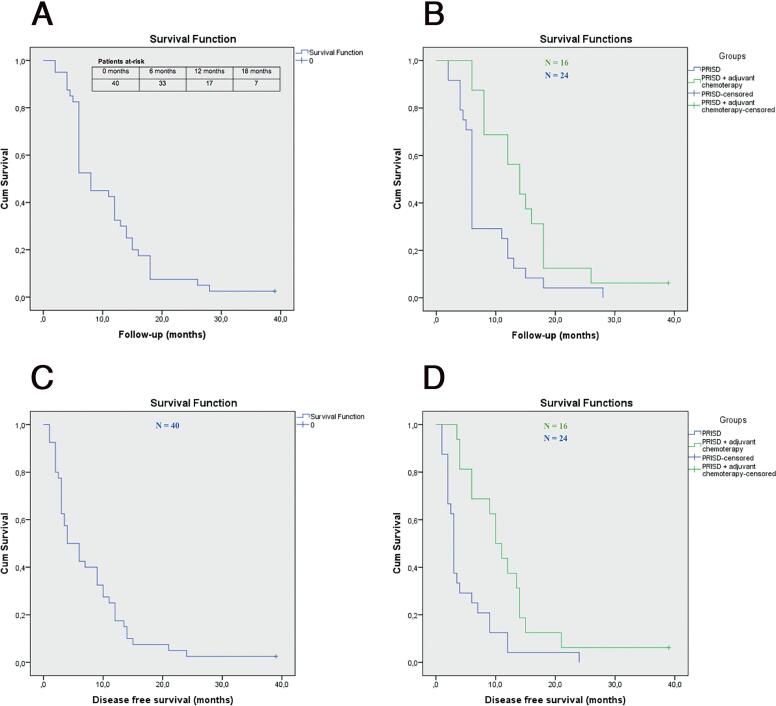
A) Kaplan-Meier plots for overall disease-specific survival. B) disease-specific survival among patients submitted to primary radical inguinal surgical debulking (PRISD) alone against PRISD with adjuvant chemotherapy. C) overall disease-free survival and D) disease-free survival among patients submitted to PRISD alone against PRISD with adjuvant chemotherapy.

All patients achieved temporary local control of the disease after PRISD with MPFR and were discharged from the hospital, achieving social and family reintegration. Table-3 summarizes patient's ECOG Performance Status, surgical treatment employed and oncological outcomes. Seven (17.5%) patients died before any adjuvant treatment could be started due to the rapid progression of the disease after the surgical procedure, 16 (40%) patients received adjuvant chemotherapy involving three courses, every 21 days, of 5-FU associated with cisplatin (continuous infusion of 5-FU 800-1000mg/m^2^/day IV on days 1-4 and cisplatin 70-80mg/m2 IV on day 1), 10 (25%) patients underwent paliative combined chemotherapy with local radiation therapy due to early disease recurrence over the MPFR, and 7 (17.5%) patients were not eligible for adjuvant chemotherapy and were referred for palliative care according to disease progression. During the follow-up, 10 (25%) patients presented local recurrence with cutaneous involvement of the MPFR, 10 (25%) patients developed loco-regional recurrences, 8 (20%) patients developed regional groin metastases in non-dissected areas, with disseminated tumor lymphadenopathy; and 11 (27.5%) patients had distant metastasis. Twenty-seven patients (67.5%) died in the first year, 10 (25%) in the second year and 2 (5%) in the third year of follow-up, with average times of 6.7, 15.9 and 27 months, respectively, leading to a DSM of 97.5%. Only 1 (2.5%) patient remained alive after 39 months of follow-up with no signs of disease. When assessing the entire sample of patients, the median cancer-specific OS was 8.0 months (95% CI 6.7-9.2) with a median DFS of 4.0 months (95% CI 1.4-6.5). The median cancer-specific OS and DFS were 6.0 months (95% CI 5.5-6.4) and 3.0 months (95% CI 2.6-3.3) respectively in 24 patients submitted exclusively to PRISD. In 16 patients who underwent PRISD with adjuvant chemotherapy, the median cancer-specific OS and DFS were 14.0 months (95% CI 10.1-17.9) and 10 months (95% CI 7.3-12.6) respectively. Statistically significant OS and DFS improvement was observed among patients who underwent PRISD with adjuvant chemotherapy compared to those who did not (p=0.006 and p=0.002). Figure-2 shows the Kaplan-Meier cumulative disease specific OS and DFS curves.

**Table 3:** Patient's characteristics and oncological outcomes in 42 patients undergoing PRISD for locally advanced penile carcinoma.

Patients Characteristics and Oncological Outcomes		Number (%)	Total
**Performance Status**			
	ECOG 2	11 (26.2)	
	ECOG 3	19 (45.2)	42*
	ECOG 4	12 (28.6)	
**Primary Tumor Treatment**			
	Partial penile amputation	24 (57.1)	
	Total penile amputation	12 (28.6)	42*
	Emasculation	6 (14.3)	
**Inguinal Lymph Node Treatment**			
	Bilateral PRISD	10 (23.8)	
	Unilateral PRISD with contra-lateral RIL	32 (76.2)	42*
**PRISD Resection Margins**			
	Negative	25 (59.5)	42*
	Positive	17 (40.5)	
**Adjuvant treatment after PRISD**			
	None	7 (17.5)	
	Adjunctive Chemotherapy	16 (40)	40⊥
	Palliative Chemotherapy with local radiation therapy	10 (25)	
	Palliative care	7 (17.5)	
**Patients Surgical Satisfaction Scale**			
	Very unsatisfied	0 (0)	
	Unsatisfied	6 (15)	
	Neither unsatisfied/satisfied	8 (20)	40⊥
	Satisfied	15 (37.5)	
	Very satisfied	11 (27.5)	
**Family Surgical Satisfaction Scale**			
Very unsatisfied		0 (0)	
Unsatisfied		4 (10)	40⊥
Neither unsatisfied/satisfied		6 (15)	
Satisfied		12 (30)	
Very satisfied		18 (45)	
**Disease Progression During Follow-Up**			
	Local recurrence with cutaneous involvement MPFR	10 (25)	
	Loco-regional recurrences	10 (25)	
	Regional groin metastases	8 (20)	40⊥
	Distant metastases	11 (27.5)	
	Free of disease	1 (2.5)	
**Disease Mortality**			
	1^st^ year	27 (67.5)	
	2^nd^ year	10 (25)	39†
	3^rd^ year	2 (5)	

ECOG = Eastern Cooperative Oncology Group; PRISD = primary radical inguinal surgical debulking; RIL = radical inguinal lymphadenectomy.

* Total number of patients submitted to PRISD;

⊥ Total number of patients on follow-up after PRISD;

† Total number of patients who evolved to death during follow-up

## DISCUSSION

The inguinal region almost invariably is the first metastasis site in PC, usually with an extended loco-regional stage before the onset of distant metastases, justifying the use of inguinal lymphadenectomy as a possible therapeutic modality. The management of advanced loco-regional disease has been changing, especially in the past 10 years, based on new evidence regarding the effectiveness of multimodal treatments. Nevertheless, the role of PRISD in patients with extensive regional metastases has received poor attention from the medical literature in recent years, with few robust reports addressing its oncological outcomes. The current guidelines of the European Association of Urology on PC (13) as well as the guidelines of the National Comprehensive Cancer Network (14) recommend neoadjuvant chemotherapy for patients eligible for cisplatinum-based regimens, with surgical consolidation in the responders, and superficial mention of the current role of PRISD in the treatment or even palliation at this stage of the disease. Brazil is a developing country with a high incidence of PC and one of the world's largest populations, of which 75% depend exclusively on the public health system for the provision of health care. With a saturated public health system and limited resources, there is great difficulty and delay for patients to access oncological treatments, especially chemotherapy and radiotherapy, justifying PRISD as a first-line treatment for patients with advanced disease.

**Figure 3:**
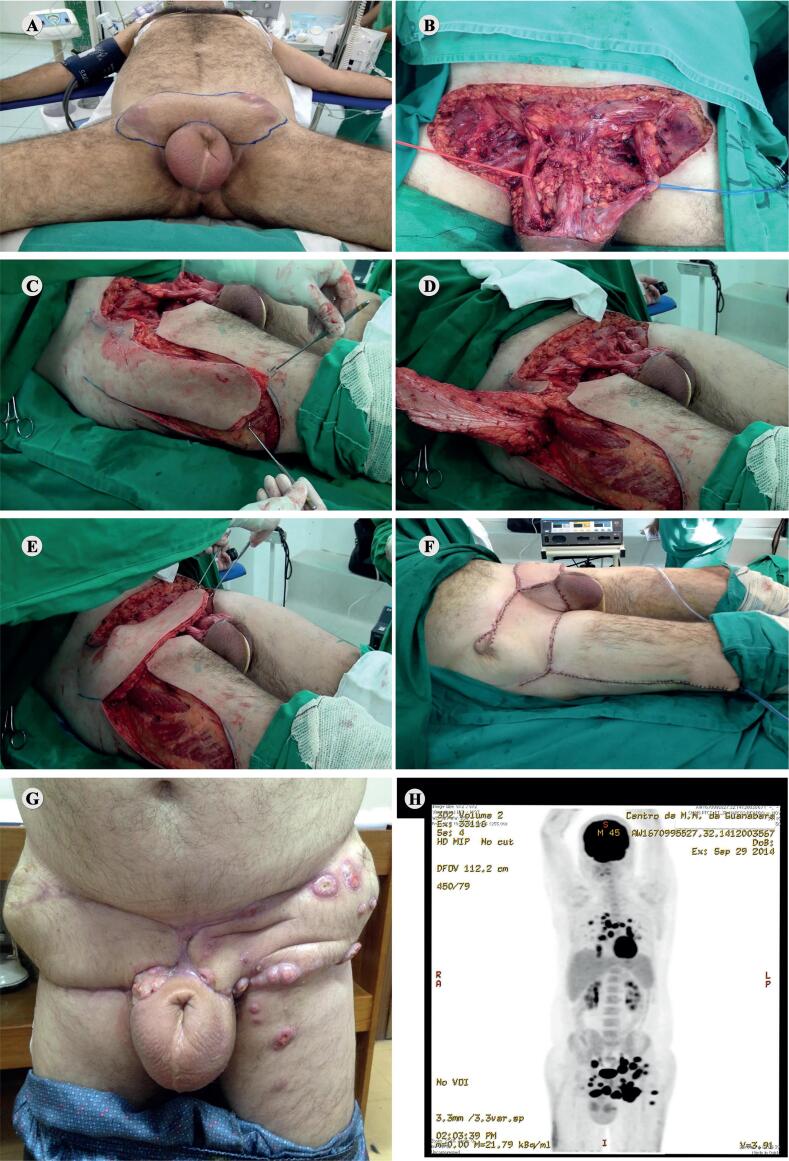
A) Patient with bulky bilateral inguinal lymphadenopathy who lost follow-up after partial penectomy, in supine position, with legs fixed in moderate external rotation, prepared for surgical approach. B) Large wound defect produced after bilateral primary radical inguinal surgical debulking (MPFR). C, D and E) Step by step bilateral MPFR using tensor fascia lata (TFL) flap. F) Final aspect of the tissue reconstruction using bilateral TFL flap. G) Local disease recurrence with cutaneous involvement of the TFL flap. H- Pet CT evidencing multiple secondary implants in the inguinal, pelvic and thoracic regions.

Surgical removal of large inguinal masses often leads to skin defects that cannot be closed primarily. Several techniques of advanced reconstructive surgery have been described to cover such defects, and the use of vascularized myocutaneous flaps is a reliable alternative to accomplish this objective (15, 16). Developing pediculate flaps requires expertise, since tissue loss can happen due to tenuous vascular supply (17). In this respect, we advocate the use of MPFR with TFL due to its ease of performance, the large amount of tissue that can be mobilized, the consistency of its vascular pedicle and the easy repair of the donor area (18). Despite this feasibility, pediculate flaps are not exempt from complications. Figure-3 shows a complete MPFR using TFL

Nirmal et al. (19) compared the morbidity between primary skin closure and TFL flap coverage of the inguinal region after standard inguinal lymphadenectomies. Eleven patients underwent 20 (9 bilateral and 2 unilateral) inguinal lymphadenectomies with primary reconstruction use of the TFL flap, with a total complication rate of 35%. In this group, 1 (5%) patient developed wound infection, 3 (15%) suffered flap necrosis and 3 (15%) seroma. In turn, Otttenhof et al. (20) published the results of 15 patients submitted to aggressive inguinal dissection due to advanced loco-regional disease with inguinal reconstruction through myocutaneous rectus abdominis or abdominal advancement flaps. They found a total complication rate of 87%, with 7 (47%) patients presenting minor complications and 6 (40%) patients suffering major complications, including 1 with DVP, 2 with abscesses and 3 patients who required additional surgery (wound necrosis debridement, flap revision and flap loss). Likewise, in the present study we found a complication rate of 88.4% related to TFL flap cover, of which 71.1% were minor and 17.3% major. Despite the high incidence of complications, most of them were minor and could be managed conservatively. Nevertheless, 2 patients developed sepsis and evolved to death while 2 surgical revisions were required for flap debridement. A possible explanation for the differences and similarities in the complication rates obtained in these studies is the heterogeneity of the disease extent and surgery among patients, besides their clinical conditions. In the present study, 31 (73.8%) patients were classified according the ECOG Performance Status as grade 3 or 4, evidencing advanced disease and limited clinical conditions. Since PC has a bilateral inguinal drainage pattern, we took care to perform contra-lateral RIL in patients who underwent unilateral PRISD in order to increase the possibilities of cure, enhance oncological results and avoid future local complications. Standard RIL was performed in 32 patients, with a complication rate of 12.5%. This result is similar to previous reports of complications published by Koifman et al. (21) and Ornellas et al. (11), supporting the excellent results of the inguinal approach using the Gibson incision.

Pelvic lymph node involvement is an independent factor of poor prognosis. Pandey et al. (22) reported that none of their 21 patients with pelvic lymph node involvement survived at least three years, as also reported by Ravi et al. (23) and Ornellas et al. (11). In the current study, 6 patients presented suspected pelvic lymph node involvement via CT scan, but none of these patients underwent pelvic lymphadenectomy. We believe that in these cases, operation is palliative, without the possibility of surgical control.

Historically, patients with bulky inguinal lymphadenopathy have had an unfavorable prognosis, regardless of the therapeutic modality chosen (24). Life expectancy in such cases is limited and depends on disease eradication, which can possibly be achieved with extensive surgery. Although negative surgical margins were achieved in 59.5% of patients in the current study, these findings did not translate into absence of loco-regional recurrence or increased survival. In this scenario, it is plausible that neoplastic cells could be present outside the resected area and micrometastases, not identified by the currently available radiological methods, are already present in other regional lymph nodes.

The available data on oncological treatments and their outcomes in treatment of advanced loco-regional disease are scarce and disappointing. Multimodal treatment with chemoradiation or neoadjuvant chemotherapy with consolidation surgery is an option advocated by some authors, despite the small number of studies published. In two retrospectives analyses, Wang et al. (25) and Pond et al. (26) reported median OS values of 12.2 and 6.9 months, respectively, after chemoradiation therapy. In a prospective study conducted by Pagliaro et al. (27), 30 patients with cN2 and cN3 PC were submitted to neoadjuvant chemotherapy with surgical consolidation and the authors found a remarkable OS of 17.1 months, with an estimated time to disease progression of 8.1 months, besides a 66.6% mortality rate. Nonetheless, the study lacked randomization, and the absence of a parallel surgical arm alone and the presence of patients with distinguished lymph node status were limitations. In contrast, in a phase 2 clinical trial, Nicholson et al. (28) investigated the tolerability and response rates to neoadjuvant chemotherapy with docetaxel, cisplatin, and 5-FU in patients with locally advanced PC, and found low response rates (36.8% cN3M0) with high intolerability to the regimen, recommending against its use in routine settings.

The current study shows overall frustrating oncological outcomes, with most of the patients evolving to death within three years of follow-up, leading to a disease-specific mortality of 97.5%. With a median overall survival of 8.0 months and a median DFS of 4.0 months, PRISD with MPFR was able to produce fleeting local control of the disease and limited survival. In a recent series published by Nicolai et al. (29), the authors retrospectively analyzed the survival rates and DFS in patients with cN2 and cN3 PC using chemotherapy in both neoadjuvant and adjuvant settings for surgery. The authors reported a two-year DFS rate of 7.1% in the neoadjuvant group against 36.8% in the adjuvant group, besides increased survival rates in the last group. Notably, in the current study, when stratifying patients according to the adjuvant therapy employed, those who received adjuvant chemotherapy showed remarkable improvements in OS and DFS, 14.0 months against 6 months (p=0.006) and 10.0 months against 3.0 months (p=0.002) respectively, compared to those who did not. The only criteria for inclusion in the current study were the presence of bulky inguinal disease and no previous inguinal treatment, without any other censoring factors, leading to a sample composed exclusively of high-risk N3 patients, with high volume disease, in contrast to the previous studies presented, which included patients with cN2 and cN3 lymph node status, with the possibility of improvements in oncological outcomes due to the presence of patients at earlier disease stage.

Most of the clinical protocols in oncology recommend the ineligibility for cisplatin-based chemotherapy of patients with ECOG Performance Status higher than grade 2 (30, 31). Although this recommendation is not an absolute contraindication of neoadjuvant or adjuvant chemotherapy, often patients with high-volume advanced disease have poor clinical conditions with high grade ECOG Performance Status, being ineligible for chemotherapy protocols, leaving surgery as the only alternative treatment. Of the 16 patients who underwent adjuvant chemotherapy in the present study, 11 (27.5%) had grade 2 and 5 (12.5%) grade 3 ECOG Performance Status respectively. The remaining 24 (60%) patients who underwent PRISD alone were classified as grade 3 or 4. Although the current study aimed to evaluate the role of PRISD in the treatment of locally advanced disease, it is possible that a large percentage of the patients involved were ineligible for chemotherapy protocols in their initial presentation.

In terms of palliation, PRISD proved to be effective, albeit temporarily, as it allowed all patients to be discharged with social and family reintegration, along with mitigation of symptoms. Although we did not assess patient's quality of life, we observed that 65% were very satisfied or satisfied with the surgical procedure, while 75% of families were very satisfied or satisfied. A plausible explanation for the percentage of surgical satisfaction reported by patients and family members is related to local control of symptoms, social reintegration and attenuation of patient care by the family.

The low incidence of PC, especially in locally advanced disease, was a limitation in this study, due to the lack of a control group and randomization. The development of a specific and validated questionnaire to assess the quality of life of patients with advanced PC is another concern and is essential for future clinical trials (32). To our knowledge, this study involves the largest sample described in the international literature on PRISD with MPFR as first-line treatment for locally advanced PC, its complications and oncological outcomes.

## CONCLUSIONS

Based on the results of the current study and the available data in the international literature, PRISD with MPFR alone should be reserved for symptomatic patients with bulky inguinal metastases, ineligible or non-responding to neoadjuvant chemotherapy, with palliation intent, as it is unlikely to promote long-term OS, although it can lead to a dramatic mitigation of local symptoms with temporary local disease control. The presence of an experienced multidisciplinary team is highly recommended due to the high incidence of surgical complications related to PRISD with MPFR. Although surgical treatment with adjuvant chemotherapy can improve OS and DFS, playing an important role in the management of patients with advanced loco regional disease, further studies are needed to determine the optimal treatment sequencing in the setting of neoadjuvant or adjuvant chemotherapy to surgery.

## ABBREVIATIONS

PRISD: primary radical inguinal surgical debulking
MPFR: myocutaneous pediculate flap reconstruction
PC: penile cancer
TFL: tensor fascia lata flap
GF: gracilis flap
RIL: radical inguinal lymphadenectomy
OS: overall survival
CT: computerized tomography
DSM: disease-specific mortality
DFS: disease free survival

## Supplementary Material

**Table:** These scales and criteria were developed by The Eastern Cooperative Oncology Group (ECOG) to assess how a patient's disease is progressing, assess how the disease affects the daily living abilities of the patient, and determine appropriate treatment and prognosis. The scale was used to clinical categorize patients pre-operatively in the current study.

ECOG PERFORMANCE STATUS*	
Grade	ECOG
0	Fully active, able to carry on all pre-disease performance without restriction
1	Restricted in physically strenuous activity but ambulatory and able to carry out work of a light or sedentary nature, e.g., light house work, office work.
2	Ambulatory and capable of all self-care but unable to carry out any work activities. Up and about more than 50% of waking hours.
3	Capable of only limited self-care, confined to bed or chair more than 50% of waking hours.
4	Completely disabled. Cannot carry on any self-care. Totally confined to bed or chair.
5	Dead.

* As published in Am. J. Clin. Oncol.:

Oken MM, Creech RH, Tormey DC, Horton J, Davis TE, McFadden ET, Carbone PP. Toxicity And Response Criteria Of The Eastern Cooperative Oncology Group. Am J Clin Oncol. 1982;5:649-55.
